# Human immuno-therapeutics for cancer treatment of dogs?

**DOI:** 10.3389/fvets.2025.1593333

**Published:** 2025-07-02

**Authors:** Hans Klingemann

**Affiliations:** Lee-Klingemann Canine Cancer Research Foundation, Boston, MA, United States

**Keywords:** immunotherapy, dogs, NK cells, lymphocytes, crossreactivity, cytokines

## Abstract

Immunotherapy for humans has enjoyed a recent boost of treatment options that, however, has not translated into the veterinary field. Developments like monoclonal antibodies against immune checkpoint inhibitors and tumor-specific CAR-T cells have broadened treatment options for human cancer patients but the canine space has not benefited from those advancements. These novel treatments are expensive to develop for the canine market and are not necessarily promising a significant financial return for the pharmaceutical industry. Hence the question is whether there are immunotherapies that work for humans and that also have some cross-species (xenogeneic) activity in dogs, but at the same time have only minimal side effects and are affordably priced. Can such an approach be considered at all assuming that the disparity could result in an immediate rejection of the administered ‘product’ with all the potential side effects? Maybe this assumption is not necessarily founded on solid data and this brief review attempts to summarize of what is actually known on the treatment of canine cancers with human immuno-therapeutics.

## Introduction

Cancer is the most common cause of death in dogs with an estimated incidence of 30–40% and increasing with age. Almost half of dogs over the age of 10 years will develop cancer and aggressive treatments (chemotherapy and radiation) are less well tolerated at that age group (1, 2).

Immunotherapy of cancer has seen some encouraging developments for humans but this has not translated to the same extent into progress for dogs. Hampel et al. (3) recently reviewed the current options and potential opportunities. Treatments like CAR-T cells, monoclonal antibodies (mAbs) and checkpoint inhibitors for canines are in their infancies and there is concern that they will ever find an accepted place in the spectrum of therapies for canine cancer (4, 5). Those treatments are expensive because of their more complex manufacturing process and lengthy path to regulatory approval. Most dog owners also have limitations as to what they can and will pay for the treatment of their beloved dog. Only some 10–15% of dog owners in the US carry health insurance for their pet and it cannot be assumed that insurance companies will pay for those more advanced and often experimental treatments. Likewise, pharmaceutical companies are not particularly motivated to invest in the development of biologics that will not promise a significant financial return. Hence immunotherapy for dogs with cancer has made limited progress (6).

There is, however, a clear need for immuno-therapeutics that are well tolerated by dogs as cancer is so much more prevalent in older dogs that tolerate chemotherapy and radiation less well (7). In fact, a recent survey suggested that about two-thirds of dog owners would not elect to treat their dog with chemotherapy or radiation due to the negative impact on their quality of life (8). Considering the limited spectrum of available immuno-therapeutics for dogs, the question is whether there are immune-active biologics that have been or are being used for *human* cancer treatment and that—because of crossreactivity—could also be used to treat cancer in dogs? In addition of the need for crossreactivity, there is also the concern that the xenogeneic cells, cytokines and biologics will induce a rejection response with neutralizing antibodies and/or xenoreactive immune cells. Such concerns go back to the data on the infusion of human serum albumin into dogs for a protein losing enteropathy (9, 10). It appears though that infusions of 5% albumin had less side effects than 25% albumin preparations. Also, a single infusion was relatively safe with anti-HLA antibodies forming only after one to two weeks. A reaction occurs mostly with the second and following infusions although it needs to be considered that about 8–10% of dogs already have antibodies against human serum albumin (11).

There are not a lot of data that would tell us how often a more severe immune reaction occurs especially in situations where there is high structural homology between human and canine proteins. Moreover, canine cancer patients, like humans, have a compromised immune reactivity which may allow for the human therapeutic to have some effect before it is “rejected.” The Toronto group has shown that the infusion of allogeneic NK cells into (human) patients with lymphoma or myeloma did not trigger HLA-antibodies in any of the 12 patients and allo-reactive T-lymphocytes (as detected in a mixed lymphocyte culture) were formed in only half of the patients (12). Even if there is a humoral and/or cellular anti-human immune response after systemic administration, could the local (intra-tumor) injection of the therapeutic be an effective alternative simply because of the higher effector concentration at the tumor site?

The objective of this review is to summarize immunotherapeutics developed for *humans* that have been given to dogs because of their potential crossreactivity. This review does not attempt to cover the entire field of immunotherapy for dogs as this has been done comprehensively by Hampel et al. (3).

## Human monoclonal antibodies

With protein-based biologics like antibodies, there are two potential considerations: *First*, is the canine target molecule at the sequence level sufficiently identical to the human equivalent for the antibody to recognize and bind the canine target antigen? S*econdly*, will the canine immune system mount an immune response against the human (xenogenic) protein that will not only result in the loss of activity but could also induce some side effects.

**Figure 1:**
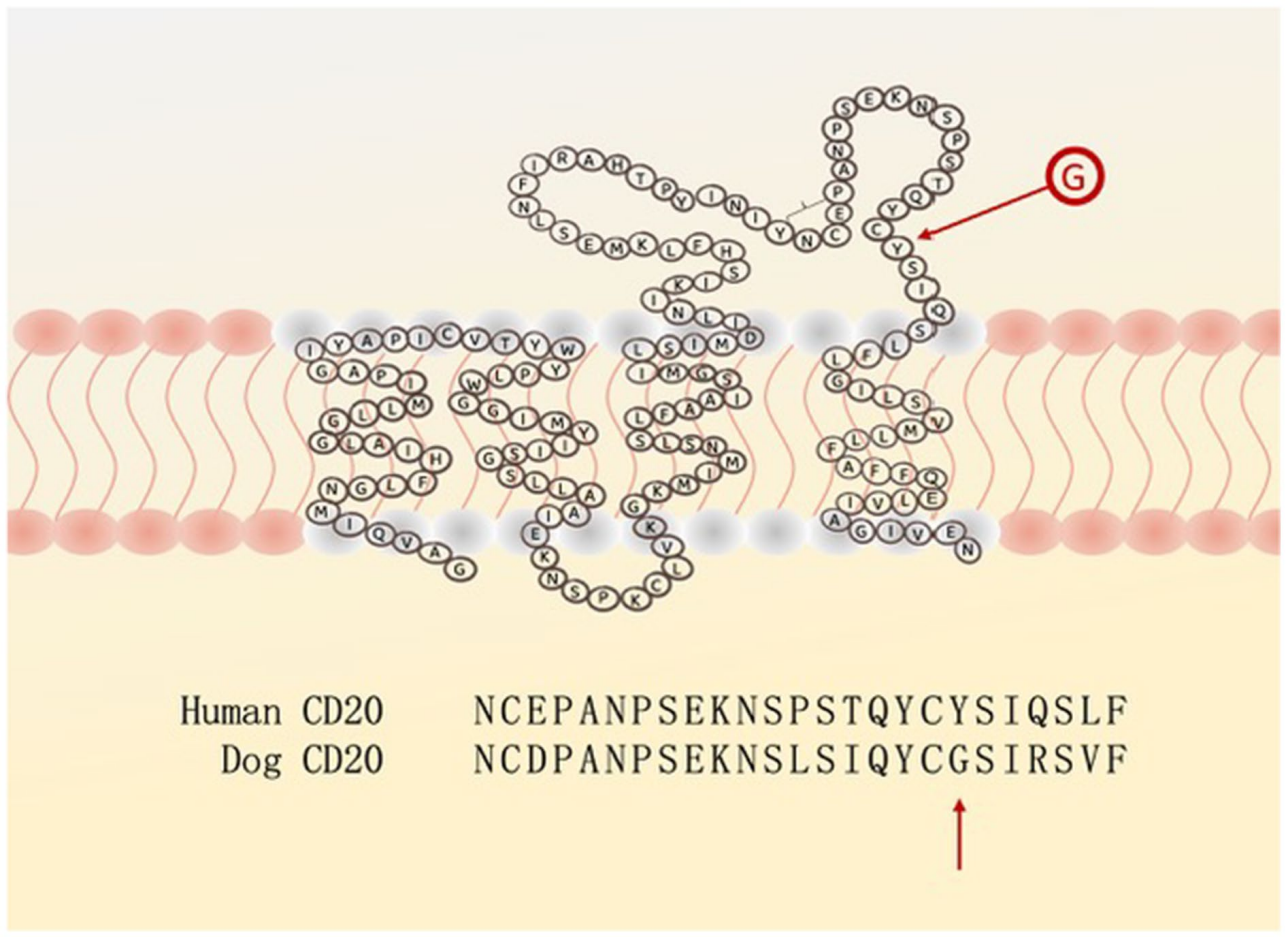
The extra-cellular loop of the CD20 surface antigen on human and canine B-cells differs in (only) one amino acid: Y (tyrosine) versus G (glycine), preventing rituximab from having its blocking activity.

As an example for the first consideration: the human anti-CD20 antibody (Rituximab^R^) is effective for the treatment of human lymphoma but a *single* amino acid difference in the CD20 binding region prevents the binding of Rituximab^R^ to the canine malignant B-cells (Figure 1) (13).

Pantelyushin et al. (14) tested seven FDA approved human immune checkpoint inhibitors targeting CTLA-4 or PD/PD-L1 against various canine tumor cell lines. Only one of the antibodies (Atezolizumab^R^) recognized the canine PD-L1 equivalent and showed some blocking effect *in vitro* of canine PBMC. Its potential clinical relevance for treatment in dogs was not further explored.

One of the main mechanisms of action for mAbs is through antibody dependent cellular cytotoxicity (ADCC) which requires an intact Fc-receptor on immune effector cells. The primary effector cells of ADCC—NK cells—are still relatively poorly described in dogs (3, 15, 16). It is also largely unknown which of the four IgG subclasses effectively mediate ADCC in dogs. Studies by Mizuno et al. (17) and Hullsiek et al. (18) have shown that the human NK cell line NK-92 can be transfected with a *canine* Fc-receptor which will allow to test canine mAb candidates for their target specificity and ADCC. Those observations also confirm that *human* perforin and granzymes are cross-reactive and kill *canine* cancer cells (19).

Even if there is some reasonable evidence for a human mAb to be cross-reactive with a specific canine cancer, the costs for such an antibody may be a significant issue for dog owners. For example, Gilvetmab^R^, a mAb against (canine) PD-1 was recently conditionally approved for treatment of melanoma and mast cell tumors in dogs. At a price of $ 1,200–1,500 per infusion (depending on the weight of the dog and the overhead charges of the practice/hospital) and the recommendation for 10 infusions, treatment costs can be substantial.

## Human T-lymphocytes

Isolating T-cells from canine blood, expanding them to significant numbers and ultimately making them tumor-specific, is a formidable challenge that so far has not translated into clinical practice. The alternative, to use T-cells from humans, has not been explored out of concern of graft-versus-host and host-versus-graft reactions. In addition, the lack of known targetable surface antigens on canine cancer cells has also stalled this potential option.

Some 30 years ago, the Philadelphia group used a broadly human cytotoxic T-cell line (TALL-104) to treat dogs with advanced cancers including osteosarcoma and histiocytosis (20, 21). In the initial trial, the cells were administered as intravenous bolus over 30 min, on alternate days for 2 weeks followed by a once weekly infusion for 3 weeks (total of nine injections) (20). Since the dogs developed anti-human antibodies after about 2 weeks into TALL-104 treatment, a follow up trial treated dogs with a modified protocol, which consisted of daily cell infusions for five consecutive days followed by monthly cell boosts (21). PCR amplification of the mini-satellite region YNZ.22 could confirm that the TALL-104 cells stayed in the dogs’ circulation for a few days after infusion.

Despite the immune response against TALL-104 cells, their effect on canine cancer was encouraging with 7/19 dogs showing some tumor regression and one dog having a complete remission. A graft-versus host reaction was not observed and none of the dogs experienced any significant side effect after the infusion. The main reason why the TALL-104 treatment did not get developed further (even for human treatment) was based on the fact that the cells were somewhat difficult to maintain in culture and to expand to numbers sufficient for multiple infusions. The study however taught us that *human* immune cells can be infused into dogs without any significant side effects and that some anti-tumor effect is achievable.

CAR-T cell therapy has enriched the treatment spectrum for human cancer patients particularly with lymphoma and myeloma. The CAR-T cell field for canine cancer treatment has been slow to develop largely due to a lack of targetable tumor antigens and low efficacy (5, 22). This has led investigators to explore *human* CAR constructs that are cross-reactive with canine antigens (14). Zhang et al. (23) reported preliminary results on *canine* CAR-T cells recognizing the *human* B7-H3 molecule. The CAR was engineered based on the sequence of the *human* mAb MGA271 (enoblituzumab) which the investigators confirmed to be cross-reactive with canine B7-H3. The cells were expanded in the presence of *human* recombinant IL-2. Two healthy beagles received lymphodepleting chemotherapy (cyclophosphamide and fludarabine) followed by infusions of B7-H4 specific CAR cells with no significant side effects.

The Flint Animal Cancer Center generated a dual CAR comprised of the *human* B7-H3 and the *human* CXCR2 sequence for canine T-cells to improve homing of those cells to osteosarcoma sites (24). For the *ex vivo* expansion and activation, the investigators used the *human* cytokines IL-2, IL-7, and IL-15 and noted that those *human* cytokines resulted in better *ex vivo* CAR-T cell generation and function than the corresponding *canine* cytokines.

## Human natural killer cells

In contrast to human natural killer (NK) cells which can be quite reliably identified as CD3 negative cells expressing the CD56 antigen, canine NK cells are not as well characterized and lack specific antigens (3, 4). The UC Davis group could characterize a subpopulation of canine lymphocytes that expresses CD5 as having NK-like activity and they were able to isolate and expand those cells from PMBC (15, 25). Clinical trials will have to show whether those cells provide an effective treatment for canine cancers. Alternatively, could there be a place for human NK cell lines, that have been immortalized and can be grown up in unlimited quantities? Although several human NK cell lines have been established (26), only NK-92 cells have consistent and broad cytotoxicity and have been given to (human) cancer patients [reviewed in Klingemann (27)]. The cells have also been genetically engineered to express a high affinity Fc-receptor, able to serve as effector cells for mAbs and with IL-2 to make their expansion independent of exogenous IL-2 (27, 28). Various CAR expressing NK-92 cell variants have been developed targeting PD-L1 and Her-2 and clinical trials in human cancer patients have been completed or are ongoing with those engineered cells (27).

To further test the extent and relevance of cross-species reactivity, NK-92 cells engineered with a murine CD20 CAR were injected into the lymphoma of immune-competent C57BL/6 mice which resulted in significantly longer survival compared to the control group (29). Likewise intra-tumor injection of (murine) Her2 + expressing CAR NK-92 cells into immunocompetent mice with glioma (GL261) resulted in tumor control and greatly extended their survival (30). Importantly, in both studies, the intra-tumor injection of CAR-specific NK-92 cells was able to induce a vaccine-like effect: when NK-92 treated mice were re-challenged with the same cancer cells after several weeks at a different body site, no tumor growth occurred.

**Figure 2:**
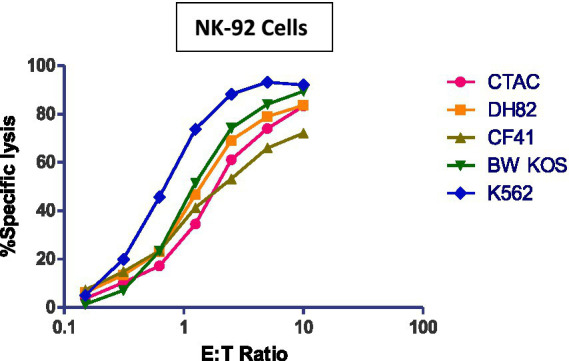
Cytotoxicity (4 h) of NK-92 cells against various canine tumor cell lines. Human K562 cells serve as positive control. Canine cell lines; CTAC: thyroid adenocarcinoma, DH82: malignant histiocytosis, CF41: mammary carcinoma, BW KOS: osteosarcoma.

NK-92 cells have cytotoxic activity against various *canine* cancer cell lines (Figure 2). Lysate generated from NK-92 cells generated by repeat freeze/thawing not only contains perforin/granzymes but also the entire spectrum of immune-active cytokines and chemokines able to control canine cancer cells (19).

## Human cytokines

### Interleukin-2

Interleukin-2 (IL-2) has been available in recombinant form for some time and has been given systemically or via intra-tumor injection to human cancer patients with documented efficacy. Since there is about 80% sequence homology between *human* and *canine* IL-2, investigators have administered the *human* version to dogs (31, 32). *Human* IL-2—as a liposomal preparation and with human serum albumin (HAS) as a solvent-was given via inhalation to seven dogs with pulmonary metastases and two dogs with lung cancer (33). Responses were encouraging with 2/4 dogs with pulmonary metastases from osteosarcoma having complete remissions lasting for more than 12 and 20 months, respectively. Those therapeutic effects were seen despite the fact that antibodies against human IL-2 and human serum albumin were detected in all dogs. Ziekman et al. (32) reported on 10 dogs with non-resectable cutaneous mastocytoma (three dogs with metastases) who were given one intra-tumor injection of 4.5 million IU of *human* IL-2 (Aldesleukin^R^). Five of seven animals with non-metastatic disease either had a partial or a complete response. Importantly, no significant side effects were reported. The same group also reported some responses in dogs with transmissible venereal tumors (TVT) after intra- and peri-tumoral injection of 2.0 million IU of *human* IL-2 (34). Of 13 dogs treated, two dogs entered a complete remission, the tumor regressed partially in one dog, and four dogs had stable disease.

Since the half life of IL-2 in the circulation is short requiring more frequent administration, the intra-tumor administration is an attractive option. Stinson et al. (35) developed a method to link IL-2 (and IL-12) to collagen which prolongs their presence in the tumor microenvironment after intra-tumor injection.

### Interleukin-12

Clinical use of this cytokine has been limited by concerns around its short half-life and narrow therapeutic index. Even when given intra-tumor, is it rapidly cleared from the tumor site requiring more frequent injections which has led to efforts to bind it in a stable complex. Options include binding interleukin-12 (IL-12) to tumor collagen (35) or “anchor” it with aluminum hydroxyte (36). *Human* IL-12 has also been administered locally by electrogene therapy (37).

The immunocytokine NHS-IL-12 consists of the heavy-chains of a *human* mAb raised against DNA released by necrostic tumor cells which is fused to two molecules of a genetically modified *human* IL-12 (38). It is currently not in trials or part of clinical practice.

### Interleukin 15 (IL-15) and IL-15:IL-15R (Anktiva^R^)

A recent study at the University of Davis administered *human* IL-15 to dogs (*n* = 21) via inhalation through a fitted nebulizer twice daily for two weeks (39). Dogs had lung lesions of their osteosarcoma or melanoma. Response rates were encouraging with stable disease (*n* = 5), partial response (*n* = 1) and one dog with a complete remission. Importantly, no side effects to the xenogeneic IL-15 were observed. As part of that study, four dogs received the *human* IL-15 superagonist Anktiva^R^ which was well tolerated.

The same group recently showed that the number of NK cells that can be obtained from unmanipulated canine PBMC is equal or even better than the yield after CD5 depletion of PBMC (16). Both fractions were expanded on an irradiated K562 feeder layer engineered to produce *human* IL-21 and *human* IL-2. Using this approach, the investigators conducted two feasibility trials with NK cells expanded from canine PBMC: *autologous* cells were injected on days 0 and 7 supported by a two week twice daily inhalation of *human* IL-15. A second trial treated dogs with advanced melanoma with *allogeneic* (canine) NK cells supported by two subcutaneous injections of *human* IL-15. No serious side effects occurred in either study and some promising responses were observed (25).

The University of Davis group is currently leading a multicenter study with inhaled *human* IL-15 in the adjuvant setting for dogs after amputation and chemotherapy for osteosarcoma [R. Rebhuhn, personal communication]. With the recent FDA approval of Anktiva^R^ for bladder cancer in humans and its cross-reactivity with canine cancer tissue, it is hoped that there will be studies testing efficacy of this unique cytokine more broadly against canine tumors.

### Hematopoietic growth factors (G-CSF and GM-CSF)

*Human* preparations for both cytokines have been administered to dogs to accelerate neutrophil recovery after chemotherapy and/or radiation. Safety and efficacy for both preparations have been confirmed (40, 41).

## Human gene engineered tumor vaccines

Gene delivery methods include both viral and non-viral vectors, such as transfer of plasmid DNA injected directly into the tumor via gene gun or electroporation. *CSPG4* is a cell surface proteoglycan overexpressed in a wide range of *human* and *canine* tumors that has been tested with the rationale to overcome unresponsiveness to “self” antigen (42). Although there was an immune response against the *human* CSPG4 in dogs with melanoma, a convincing benefit of this treatment has not been shown (43). The same is true for intradermal injection of *human* tyrosinase DNA in a bacterial plasmid (Oncept^®^). It is suggested to induce a humoral and cytotoxic T-cell response against canine melanoma cells that express tyrosinase (44). The product is USDA—approved for the treatment of dogs with stage II or III oral melanoma. Although a popular therapy among veterinarians, its efficacy has not conclusively shown in well-designed clinical trials (45).

Oncolytic viruses are receiving some attention as they could make tumors more immunogeneic or modulate the tumor microenvironment. Studies in humans with different viruses are ongoing (46) T-VEC, also known as Imlygic^R^, is a genetically modified herpes simplex virus, approved by the FDA for the treatment of unresectable melanoma in humans (47). T-VEC has not been studied yet in canines although administration of some experimental oncolytic viruses (i.e., adenovirus based) have suggested the occasional benefit (48). One challenge in using oncolytic viruses in dogs is the potential for neutralizing antibodies against the virus, especially if the dog has been vaccinated against a similar virus.

## Conclusion

Veterinarians should not be overly concerned that a human (xenogeneic) immune-based therapeutic will cause significant side effects in dogs or may be ineffective because of an immediate or delayed immunological rejection. Especially the *intra-tumor* injection or *inhalation* of a human cytokine (IL-2, IL-12, IL-15) could be an attractive treatment option for certain canine cancers with minimal side effects and the possibility of inducing a vaccine-like effect. Cytokines offer the opportunity to be combined with radiation or low dose chemotherapy as recently shown for IL-2 and IL-12 (49) with the rationale to induce local tumor cell apoptosis/necrosis and exposing tumor antigens to become targets for cytokine activated lymphocytes. The costs for such treatment protocols should be reasonable compared to the more significant costs of monoclonal antibodies or cell-engineered treatments. A general challenge though in immunotherapy trials in dogs and arriving at conclusions is the widespread use of corticosteroids in veterinary practices which can make it difficult to assess the contribution of a specific therapeutic (50).
